# Advancing free‐breathing liver diffusion‐weighted imaging with Propeller‐EPI: Improved image quality and ADC repeatability

**DOI:** 10.1002/mp.70180

**Published:** 2025-12-04

**Authors:** Liyuan Liang, Lu Wang, Qiting Wu, Guangtao Chen, Keith Wan‐Hang Chiu, Yi‐Jui Liu, Chang‐Hsien Liu, Chun‐Jung Juan, Yat Lam Wong, Hailin Xiong, He Wang, Hsiao‐Wen Chung, Hing‐Chiu Chang

**Affiliations:** ^1^ Department of Biomedical Engineering The Chinese University of Hong Kong Hong Kong SAR China; ^2^ Multi‐Scale Medical Robotics Center Hong Kong SAR China; ^3^ Department of Diagnostic Radiology The University of Hong Kong Hong Kong SAR China; ^4^ College of Information Science and Engineering Northeastern University Liaoning China; ^5^ Department of Health Technology and Informatics The Hong Kong Polytechnic University Hong Kong SAR China; ^6^ School of Chinese Medicine The Chinese University of Hong Kong Hong Kong SAR China; ^7^ Department of Diagnostic and Interventional Radiology Queen Elizabeth Hospital Hong Kong SAR China; ^8^ Department of Automatic Control Engineering Feng Chia University Taichung Taiwan; ^9^ Department of Medical Imaging China Medical University Hsinchu Hospital and China Medical University Hsinchu Taiwan; ^10^ Department of Radiology Tri‐Service General Hospital and National Defense Medical Center Taipei Taiwan; ^11^ Institute of Nuclear Engineering and Science National Tsing Hua University Hsinchu Taiwan; ^12^ Department of Medical Imaging China Medical University Hospital Taichung Taiwan; ^13^ Department of Radiology, School of Medicine China Medical University Taichung Taiwan; ^14^ Department of Biomedical Engineering National Defense Medical Center Taipei Taiwan; ^15^ Department of Computer Science and Information Engineering National Taiwan University Taipei Taiwan; ^16^ Department of Medical Imaging China Medical University Hsinchu Hospital Hsinchu Taiwan; ^17^ Department of Clinical Oncology Queen Mary Hospital Hong Kong SAR China; ^18^ Institute of Science and Technology for Brain‐Inspired Intelligence Fudan University Shanghai China; ^19^ Key Laboratory of Computational Neuroscience and Brain‐Inspired Intelligence (Fudan University) Ministry of Education Shanghai China; ^20^ Human Phenome Institute Fudan University Shanghai China; ^21^ Graduate Institute of Biomedical Electrics and Bioinformatics National Taiwan University Taipei Taiwan

**Keywords:** Diffusion‐weighted imaging, free‐breathing, liver ADC measurement, liver DWI, multi‐shot DWI, Propeller‐EPI

## Abstract

**Background:**

Liver diffusion‐weighted imaging (DWI) with apparent diffusion coefficient (ADC) measurement has proven valuable in diagnosing liver diseases. In certain patient populations, a free‐breathing (FB) liver DWI approach is desirable to improve patient comfort and broaden clinical applicability. However, maintaining high image quality under FB conditions and achieving satisfactory ADC repeatability can be challenging when using routine diffusion‐weighted single‐shot echo‐planar imaging (DW‐ss‐EPI).

**Purpose:**

This study aimed to develop an advanced FB liver DWI technique based on diffusion‐weighted Propeller echo‐planar imaging (DW‐Propeller‐EPI) to achieve motion compensation, and to prospectively evaluate its performance in terms of image quality and ADC repeatability compared to standard DW‐ss‐EPI.

**Methods:**

A DW‐Propeller‐EPI pulse sequence and reconstruction pipeline was developed on a 1.5T MRI system. The pipeline incorporated a reference‐free Nyquist ghost correction method and motion compensation using correlation weighting during data reconstruction. For in vivo evaluation, participants prospectively underwent two repeated liver DWI scans using four techniques: three routine DW‐ss‐EPI sequences with different breathing controls (breath‐holding, BH; respiratory‐triggering, RT; and FB), and one FB DW‐Propeller‐EPI sequence. The raw data from the FB DW‐Propeller‐EPI scans were processed offline with the developed motion‐compensated reconstruction. Two radiologists independently rated the image quality on a 5‐point scale across five aspects (signal homogeneities in the left lobe, geometric fidelity, liver edge sharpness, vessel clarity, and overall image quality). Mean scores were compared among the four techniques using the Friedman test. The repeatability of ADC measurements was evaluated with Bland‐Altman analysis, and differences in ADC values between liver lobes and techniques were analyzed using two‐way repeated measures ANOVA.

**Results:**

35 participants were enrolled, with twenty completing repeated scans. FB DW‐Propeller‐EPI demonstrated significantly higher ratings than the three routine DW‐ss‐EPI sequences across all aspects (all *P* < 0.001), with good interobserver agreements (ICC ranging from 0.80‐0.88). The mean geometric fidelity score of FB DW‐Propeller‐EPI was rated the highest (4.86 ± 0.33). FB DW‐Propeller‐EPI also exhibited superior ADC repeatability in both the left and right liver lobes (LOA: 0.212 × 10^−3^ mm^2^/s versus 0.255 × 10^−3^ mm^2^/s). ADC measurements were comparable between FB DW‐Propeller‐EPI and both RT or FB DW‐ss‐EPI techniques.

**Conclusions:**

The proposed FB DW‐Propeller‐EPI technique enables high‐quality FB liver DWI with satisfactory ADC repeatability, outperforming conventional DW‐ss‐EPI in image quality and ADC repeatability. This advancement addresses the limitations of routine DW‐ss‐EPI in FB scenarios, offering a promising solution for clinical applications in challenging population.

## 1 INTRODUCTION

Liver diffusion‐weighted imaging (DWI) with apparent diffusion coefficient (ADC) measurement has shown notable potential in detecting and characterizing liver lesion^1^, monitoring treatment response^2^, and assessing liver fibrosis or cirrhosis.^3^, ^4^ Accurate and repeatable liver DWI is crucial for timely diagnosis and monitoring of liver diseases. However, subject motion and the use of diffusion‐weighted single‐shot echo‐planar imaging (DW‐ss‐EPI) as the imaging technique can both decrease the image quality and ADC repeatability.^5^, ^6^ These limitations may hinder the application of liver DWI due to inconsistent image quality.

Over the past decade in routine liver DWI practice, various breathing schemes have been employed with DW‐ss‐EPI to mitigate the effects of respiratory motion. While breath‐holding (BH) liver DWI acquire data within a short duration to mitigate respiratory motion, this rapid acquisition can compromise image quality, resulting in limited resolution and inferior signal‐to‐noise ratio (SNR) compared to longer scan time.^7^ This method is particularly useful for minimizing total examination time. In contrast, respiratory‐triggering (RT) liver DWI enables multiple signal averaging with longer scan duration, offering a better tradeoff between image quality and acquisition efficiency.^7^ However, both BH and RT DW‐ss‐EPI may not be suitable for challenging populations with limited BH capacity or irregular respiration, potentially leading to prominent artifacts or extended scan durations that can affect ADC measurement accuracy. As an alternative, free‐breathing (FB) liver DWI is desirable for these populations. While some studies have suggested that FB acquisition may improve the repeatability of ADC measurement,^7^ other work has indicated poor ADC repeatability with FB approaches.^5^, ^8^, ^9^ This discrepancy may be partially attributed to variations in image quality achieved by FB liver techniques. Specifically, current FB liver DWI often suffers from image blurring and reduced organ sharpness due to signal averaging of misregistered data.^6^, ^10^

In addition to respiratory motion, using DW‐ss‐EPI for liver data acquisition also leads to degraded image quality due to image blurring, geometric distortion from susceptibility effects^11^ and EPI Nyquist ghost artifact.^12^ Parallel imaging techniques may partially alleviate the susceptibility artifacts, but at the cost of amplified noises and decreased SNR.^13^ Furthermore, cardiac motions can exacerbate signal loss and affect the repeatability of liver ADC measurements, particularly in the left liver lobe.^6^ Consequently, both respiratory and cardiac motions, along with the inherent limitations of DW‐ss‐EPI, contribute to decreased image quality and instability in ADC measurements for FB liver DWI. Therefore, there is a compelling need for an advanced imaging technique that is less susceptible to motions and capable of providing robust ADC measurement, particularly for patients unable to reliably perform breath‐holds or maintain regular respiration.

Promising approaches to address these challenges include multi‐shot EPI and Propeller acquisition techniques.^14^, ^15^ Various multi‐shot EPI methods have been proposed to achieve a better trade‐off between scan speed and image quality for DWI applications.^15^, ^16^, ^17^ A recent study also demonstrated that multiplexed sensitivity encoding (MUSE) can efficiently address inter‐shot inconsistencies and can be combined with RT to improve the quality of liver DWI.^15^, ^18^ On the other hand, the Propeller technique, initially developed for fast spin‐echo (FSE) imaging, has demonstrated its capability to correct or compensate for both voluntary and involuntary motions in routine FSE imaging for brain^14^, ^19^, ^20^ and body^21^, ^22^, ^23^ applications. Building on this, combining Propeller‐FSE with RT has shown potential to reduce image artifacts and improve liver edge sharpness in liver DWI.^21^, ^22^ However, the use of Propeller‐FSE may be limited by its high RF specific absorption rate (SAR) due to multiple refocusing RF pulses, particularly at high magnetic fields.^24^ Alternatively, by combining Propeller acquisitions with multi‐shot EPI, Propeller‐EPI inherits the capability of motion compensation and offers an improvement in achievable image quality,^25^ while also mitigating the SAR issue associated with FSE acquisition.^24^ Propeller‐EPI has demonstrated its potential to simultaneously improve motion immunity and geometric fidelity in various brain applications,^24^, ^25^, ^26^, ^27^, ^28^, ^29^ such as the diffusion‐weighted Propeller‐EPI (DW‐Propeller‐EPI) used for high‐resolution diffusion‐tensor imaging.^24^, ^26^ Accordingly, it is plausible that DW‐Propeller‐EPI may enable FB liver DWI with improved image quality compared to routine DW‐ss‐EPI. Nevertheless, the EPI readout used for rotating multi‐blade acquisitions intrinsically requires two‐dimensional (2D) Nyquist ghost correction. In FB liver imaging, respiratory and cardiac motions can further compromise the effectiveness of reference‐based correction methods, necessitating a reference‐free 2D approach.

The purpose of this study was to develop an advanced FB liver DWI technique based on DW‐Propeller‐EPI and evaluate its potential to improve image quality and ADC repeatability. We aimed to compare it with routine BH, RT, and FB liver DW‐ss‐EPI techniques in patients with normal breath‐holding capacity using a 1.5T MRI scanner.

## 2 METHODS

### 2.1 Data acquisition and reconstruction for FB liver DW‐Propeller‐EPI

**FIGURE 1:**
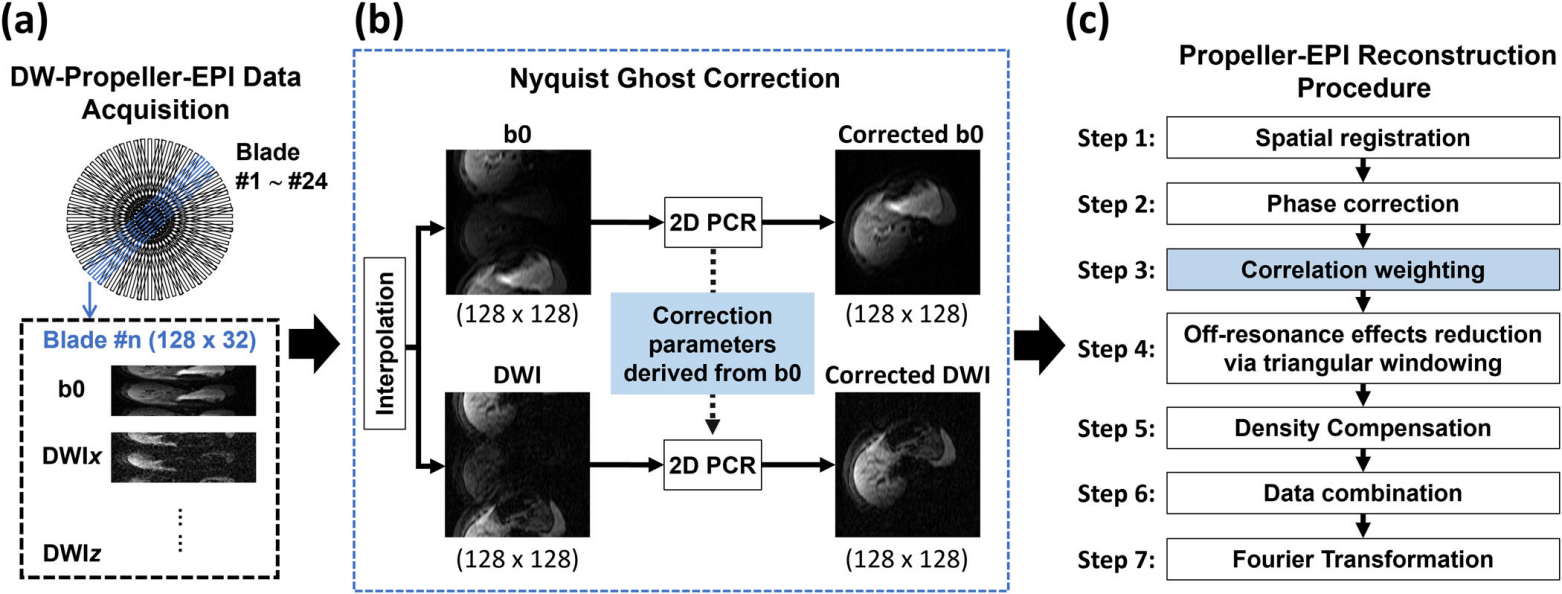
Flowchart illustrates the developed diffusion‐weighted Propeller echo‐planar imaging (DW‐Propeller‐EPI) acquisition and reconstruction scheme. (a) Free‐breathing liver DW‐Propeller‐EPI data were acquired with 24 blades (blade size: 128× 32, and rotating angle: 15°) for each diffusion direction (i.e., b‐values of 0 and 500 s/mm^2^ with three orthogonal diffusion directions). (b) The multi‐blade data were interpolated and then underwent Nyquist ghost correction using the 2D phase‐cycled reconstruction (PCR) method. For the blades acquired with the same rotating angle, the ghost correction parameters were first derived from the data acquired at b‐values of 0 s/mm^2^, and then applied to correct the data acquired at b‐value = 500 s/mm^2^. (c) For each slice location at each diffusion direction, ghost‐corrected multi‐blade data underwent the Propeller‐EPI reconstruction procedure with motion compensation. In Step 3, the k‐space data of each blade were weighted by the correlation measured between each blade image itself and the reference image (i.e., average of all multi‐blade images), to further reduce the effects of motion artifacts.

FB liver DW‐Propeller‐EPI data were acquired from participants during free breathing using a home‐built long‐axis DW‐Propeller‐EPI pulse sequence.^24^ 24 blades, each with a size of 128 × 32 and rotating angle of 15°, were consecutively acquired to achieve 360° coverage of k‐space data for each image (Figure 1a). A modified DW‐Propeller‐EPI reconstruction pipeline has been developed to produce FB liver DW images with minimal artifacts from the acquired k‐space data. This modified pipeline includes two main reconstruction modules: Nyquist ghost correction (Figure 1b) and Propeller‐EPI data reconstruction (Figure 1c).

In the first module, prior to Nyquist ghost correction, the data for each blade was interpolated to a 128 × 128 image via zero‐padding. Because the rotating blades in DW‐Propeller‐EPI require effective 2D Nyquist ghost correction without complex calibrations and with robustness to motion, a reference‐free 2D phase‐cycled reconstruction (PCR) method^30^ was adopted. For each blade acquired with a specific rotating angle, ghost correction parameters were derived directly from data acquired at a b‐value of 0 s/mm^2^ by performing the following steps:

- The zero‐padded k‐space was decomposed into two subsets (odd and even echoes), each of which was Fourier transformed into an aliasing image.
- To enhance motion robustness, a 2D linear model with a constant phase error term was used as a constraint to initiate phase cycling for searching the correction parameters for low‐quality blade images (e.g., presence of signal void due to motion or susceptibility effect).
- To ensure adequate correction across different rotating angles, the maximum potential 2D linear phase error was set to ± 2π, generating a total of 2,500 different 2D phase error profiles.
- Following the original PCR method, the two aliasing images were used to form a set of linear systems corresponding to the 2,500 2D phase error profiles.
- Each linear system was solved to generate 2,500 ghost‐corrected images, and the background energy of each image was measured.
- The ghost‐corrected image with the lowest background energy was selected as the optimal correction result, and the corresponding 2D phase error profile was applied to the data acquired at a b‐value of 500 s/mm^2^.

In the second module, for each slice at each acquired diffusion direction, the corresponding ghost‐corrected multi‐blade data underwent the Propeller‐EPI reconstruction procedure shown in Figure 1c. This procedure includes applying a set of correlation weightings to the multi‐blade data for further motion compensation (Step 3 in Figure 1c). The correlation weighting for each blade was measured as the correlation between that blade image itself and the reference image (i.e., average of all multi‐blade images), minimizing the motion‐induced signal variations across multi‐blade data. Afterward, density compensation (Step 5 in Figure 1c) incorporated both triangle weighting, to reduce the off‐resonance effect, and correlation weighting. This ensured a uniform signal sampling density after combining all blades in k‐space (Step 6 in Figure 1c). The final image was reconstructed using the direct Fourier transform of the combined k‐space data. For each slice, the mean diffusion image was produced by averaging the reconstructed images from three orthogonal diffusion directions.

**FIGURE 2:**
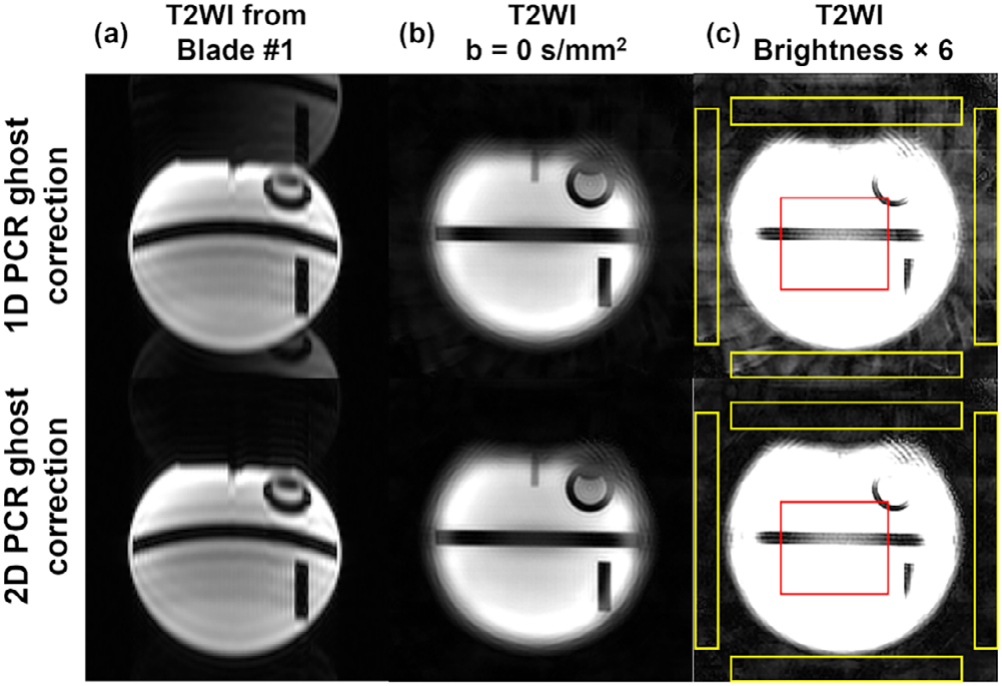
Phantom images reconstructed from the DW‐Propeller‐EPI reconstruction pipeline using either 1D or 2D phase‐cycled reconstruction (PCR) ghost correction adopted in the Nyquist ghost correction module. (a) Ghost corrected T2WI images from the first blade. (b) T2WI images reconstructed from the pipeline. (c) Reconstructed T2WI images with intensity rescaled six times. The 2D PCR method showed more effectively mitigated ghost artifacts than the 1D approach. The exhibited residual ghost artifacts in individual blades from the 1D PCR ghost correction contributed to the elevated background noise in the reconstructed images. Ghost‐to‐signal ratios (GSR) were quantitatively measured as the ratio of average intensity in four ghost regions of interest (the yellow rectangular ROIs) to a central ROI (the red rectangular ROI). GSR were 5.60% and 2.36% for 1D and 2D PCR ghost correction, respectively.

Figure 2 demonstrates the phantom images reconstructed from the above pipeline using either 1D or 2D PCR ghost correction adopted in the Nyquist ghost correction module, illustrating the effectiveness of the 2D reference‐free PCR method in minimizing residual ghost artifacts.

### 2.2 Study participants and scan protocol for evaluation

This prospective study was approved by our institutional ethic committee and written informed consent was obtained from each participant. Between June 2020 and June 2021, patients with metabolic dysfunction who were referred to our institution for liver fat assessment using non‐contrast MRI were randomly recruited for this study. The inclusion criteria were: a) aged over 18 years; b) no pregnancy; and c) ability to hold their breath for 20 s. The exclusion criteria were: a) previous liver surgery and b) failure to complete all imaging sequences for any reason.

All participants were examined on a 1.5 T clinical MRI scanner (Signa Explorer, GE Healthcare, Waukesha, WI, USA) with a 12‐channel phase‐array body coil. Liver DWI data were acquired with four techniques, including three routine DW‐ss‐EPI sequences with different breathing schemes (i.e., BH, RT, and FB), and the FB DW‐Propeller‐EPI sequence. The scan time for BH DW‐ss‐EPI, FB DW‐ss‐EPI, and FB DW‐Propeller‐EPI sequences were 18, 68, and 388 s, respectively, and the median scan time for RT DW‐ss‐EPI sequence was 145.71 s. All DWI images were acquired in the axial plane with three orthogonal diffusion directions and two b‐values (0 and 500 s/mm^2^). For the assessment of ADC repeatability, two scans were performed for each technique using identical parameters. Pulse sequences were added during the patient's clinical examination, with an additional time of around 25–30 min with two repetitions. In addition to DWI data acquisition, the axial BH fast gradient‐echo (FGRE) imaging with identical geometric parameters was also performed on each subject to provide a gold standard reference of geometric fidelity. Detailed scan parameters of all sequences are summarized in Table 1.

**TABLE 1:** Imaging parameters for BH DW‐ss‐EPI, RT DW‐ss‐EPI, FB DW‐ss‐EPI, FB DW‐Propeller‐EPI, and FGRE.

Parameters	BH DW‐ss‐EPI	RT DW‐ss‐EPI	FB DW‐ss‐EPI	FB DW‐Propeller‐EPI	FGRE
Field of view, mm^2^	380 × 380	380 × 380	380 × 380	380 × 380	380 × 380
Recon Matrix	128 × 128	128 × 128	128 × 128	128 × 128	128 × 128
Blade size	—	—	—	128 × 32	—
Receiver bandwidth, kHz	± 250	± 250	± 250	± 250	± 31.25
Repetition time, ms	2000	5454‐17142	4000	4000	160
Echo time, ms	68.4	65.3	65.3	66.4	4.2
Echo spacing, ms	0.672	0.676	0.676	0.672	—
PF factor	0.625	0.625	0.625	1	—
Parallel imaging factor	1	1	1	1	1
Slice thickness, mm	8	8	8	8	8
No. of signals acquired	2	4	4	1	1
b values, s/mm^2^	0, 500	0, 500	0, 500	0, 500	—
Scan time, s	18	92.72‐291.41	68	388	16.67
Fat suppression	Water‐excitation SPSP	Water‐excitation SPSP	Water‐excitation SPSP	Water‐excitation SPSP	—

Abbreviations: BH DW‐ss‐EPI, breath‐holding diffusion‐weighted single‐shot echo‐planar imaging; FB DW‐Propeller‐EPI, free‐breathing diffusion‐weighted Propeller echo‐planar imaging; FB DW‐ss‐EPI, free‐breathing diffusion‐weighted single‐shot echo‐planar imaging; FGRE, fast gradient‐echo; PF factor, Partial Fourier factor, which indicates the fraction of k‐space lines acquired in the phase‐encoding direction; RT DW‐ss‐EPI, respiratory‐triggering diffusion‐weighted single‐shot echo‐planar imaging; SPSP, Spectral‐Spatial.

### 2.3 Data reconstruction and image analysis

The raw data from FB DW‐Propeller‐EPI acquisitions and the DICOM images of three routine DW‐ss‐EPI techniques were transferred to a workstation PC (2.71 GHz Intel Core i5 CPU; 8 GB DDR4 memory) for offline image reconstruction and the calculation of ADC maps, using Matlab (MathWorks, Natick, MA, USA). The reconstruction pipeline for DW‐Propeller‐EPI, as described earlier, was implemented to produce averaged DWI images from the raw data.

For qualitative evaluation, the DWI images (acquired at a b‐value of 500 s/mm^2^) produced by the four imaging techniques were rated by two independent radiologists (with 32 and 34 years of experience in abdominal MRI interpretation, respectively) across five aspects: signal homogeneity in the left lobe, geometric fidelity, liver edge sharpness, vessel clarity, and overall image quality, using a 5‐point Likert scale (Table 2). The images from the four techniques were exported as files in the Enhanced Metafile (EMF) format using Matlab, generating four sets of files for subsequent review. Prior to the visual assessment, the image sets were randomly ordered for each participant. After randomization, the raters reviewed the images on a workstation PC (2.71 GHz Intel Core i5 CPU; 8 GB DDR4 memory). Both raters were blinded to the imaging techniques, scan parameters, and participant demographics.

**TABLE 2:** Image Quality Scoring Criteria for DWI (b = 500 s/mm^2^) images acquired from four techniques.

Aspects	Score = 5	Score = 4	Score = 3	Score = 2	Score = 1
Signal homogeneity in the left liver lobe	Excellent –No visible signal loss	Good –Slight signal loss	Moderate signal loss	Severe signal loss	Left liver lobe barely identifiable
Geometric fidelity	No distortion	Mild distortion	Moderate distortion	Severe distortion	Nondiagnostic
Liver edge sharpness	Sharp	Mild blurring	Moderate blurring	Severe blurring	Nondiagnostic
Vessel clarity	No blurring	Mild blurring	Moderate blurring	Severe blurring	Nondiagnostic
Overall image quality	Excellent	Good and not affecting interpretation	Moderate and potentially affecting interpretation	Poor and definitely affecting interpretation	Nondiagnostic

*Note*: The 5‐point Likert scale was used for scoring image quality.

For quantitative evaluation, ADC values were measured in both left and right liver lobes on a representative central slice, selected at the level of the main stem of right portal vein. The left and right lobes were divided by the middle hepatic vein, and measurements were performed simultaneously. Three circular regions of interest (ROIs), each with an approximate area of 100 mm^2^, were placed in each liver lobe with the exclusion of visible vessels, tissue boundaries, and severe signal inhomogeneity. Subsequently, the ADC value of each liver lobe was calculated by averaging the values from all three ROIs. For assessing ADC repeatability, ROIs were placed in similar locations on the images from two repeated acquisitions.

## 3 EVALUATION OF THE NUMBER OF BLADES IN FB DW‐PROPELLER‐EPI

### 3.1 Phantom study for image quality assessment

To investigate the influence of the number of blades on image quality of DW‐Propeller‐EPI, phantom experiments were performed using the home‐built long‐axis DW‐Propeller‐EPI sequence. 10 datasets were acquired with five different numbers of blades (8, 12, 16, 20, and 24 blades), and two k‐space coverage schemes (180° and 360°). DWI images with one diffusion direction and two b‐values (0 and 500 s/mm^2^) were acquired for each dataset. All acquisitions were performed with the following parameters: reconstruction matrix = 128 × 128, FOV = 240 mm, slice thickness = 5 mm, and TR = 4000 ms. Blades were evenly distributed in k‐space for each acquisition. The rotating angles and corresponding blade sizes for each acquisition are summarized in Supplementary Table S1. Image reconstruction was performed using the DW‐Propeller‐EPI reconstruction pipeline described in Figure 1.

### 3.2 Effects of the number of blades on ADC repeatability

The impact of the number of blades on ADC repeatability was evaluated using FB liver DW‐Propeller‐EPI data included in this study. The 24‐blade FB DW‐Propeller‐EPI datasets (i.e., two repetitions) were retrospectively reconstructed with varying numbers of blades to produce additional 12 datasets. With a fixed rotating angle of 15°, the number of blades was incrementally increased from 12 to 23 in a sequential manner, corresponding to k‐space coverage ranging from 180° to 345° in 15° increments. These retrospective reconstructions yielded 12 additional image sets for each included subject beyond the original 24‐blade dataset. For quantitative evaluation, ROIs drawn on the 24‐blade dataset were applied to measure ADC repeatability and SNR for the original and additional 12 datasets. SNR was computed by dividing the average DWI signal from the first scan by the standard deviation of the difference between the two scans.

### 3.3 Statistical analysis

Statistical analyses were performed using SPSS (IBM, version 27.0; SPSS, Chicago, Ill) and a *P* < 0.05 was considered statistically significant. Interobserver agreement for qualitative ratings was assessed using intraclass correlation coefficients (ICCs). An ICC value below 0.5, between 0.5 and 0.75, and greater than 0.75 indicated poor, moderate, and good agreement, respectively. Differences in qualitative ratings among the four techniques were compared using the Friedman test. Differences in ADC measurements between the two liver lobes and among the different techniques were compared using a two‐way repeated measures ANOVA. Post‐hoc pairwise comparisons were performed for both the Friedman test and the two‐way repeated measures ANOVA using a Bonferroni correction. Repeatability of ADC measurements was assessed using Bland‐Altman analysis. For each of the four imaging techniques, the mean difference (bias) between repeated measurements and the limits of agreement (LOA) (i.e., 95% confidence interval of the mean difference between two scans) were calculated. Linear regression was used to examine the association between the number of blades and measured ADC repeatability (LOA), as well as the association between the number of blades and SNR. The coefficient of determination (R^2^) was computed to assess the goodness of fit for both associations.

## 4 RESULTS

### 4.1 Participant characteristics

There were 43 participants enrolled in this study, and eight were excluded due to previous liver surgery (*n* = 5) or incomplete examinations (*n* = 3) (see Supplementary Figure S1 for the study inclusion and exclusion flowchart). In total, 35 participants successfully completed the acquisition of the four imaging techniques (24 men, 11 women; mean age, 46 years ± 15; range, 22–72 years). Because of limited scan time, only 20 participants underwent repeated acquisitions of all four imaging techniques for assessing ADC measurement repeatability (12 men, 8 women; mean age, 41 years ± 15; range, 22–66 years; see Supplementary Table S2 for demographic characteristics).

### 4.2 Qualitative evaluation of image quality

Qualitative ratings, interobserver agreements, and statistical significance are summarized in Table 3, with post‐hoc pairwise comparison results detailed in Supplementary Table S3. The Friedman test indicated significant differences among the four techniques regarding all five image quality rating aspects (Table 3). Post‐hoc pairwise comparisons revealed that FB DW‐Propeller‐EPI yielded significantly higher scores in all rating aspects compared to the three routine DW‐ss‐EPI techniques (Supplementary Table S3). These qualitative ratings demonstrated good interobserver agreements (ICC ranging from 0.80‐0.88, as shown in Table 3). Additionally, among the three routine DW‐ss‐EPI techniques, RT DW‐ss‐EPI yielded significantly higher scores than FB DW‐ss‐EPI for vessel clarity (3.33 ± 0.72 versus 2.49 ± 0.61) and overall image quality (3.26 ± 0.53 versus 2.53 ± 0.66), with specific comparison details in Table 3 and Supplementary Table S3.

**TABLE 3:** Qualitative image quality ratings and the interobserver ICCs between two raters.

	BH DW‐ss‐EPI		RT DW‐ss‐EPI		FB DW‐ss‐EPI		FB DW‐Propeller‐EPI		P value†
Rating Aspects	Mean ± SD	ICC	Mean ± SD	ICC	Mean ± SD	ICC	Mean ± SD	ICC	
Signal homogeneity in the left liver lobe	3.04 ± 0.73	0.66 (0.32, 0.83)	2.97 ± 0.69	0.70 (0.41, 0.85)	2.59 ± 0.75	0.76* (0.52, 0.88)	4.56 ± 0.57	0.85* (0.69, 0.92)	<0.001
Geometric fidelity	2.80 ± 0.70	0.78* (0.56, 0.89)	3.40 ± 0.67	0.80* (0.62, 0.90)	3.07 ± 0.68	0.80* (0.60, 0.90)	4.86 ± 0.33	0.88* (0.75, 0.94)	<0.001
Liver edge sharpness	3.29 ± 0.62	0.67 (0.35, 0.84)	3.06 ± 0.57	0.64 (0.29, 0.82)	2.83 ± 0.66	0.68 (0.37, 0.84)	4.69 ± 0.46	0.82* (0.65, 0.91)	<0.001
Vessel clarity	3.17 ± 0.75	0.72 (0.45, 0.86)	3.33 ± 0.72	0.76* (0.53, 0.88)	2.49 ± 0.61	0.71 (0.42, 0.85)	4.60 ± 0.51	0.80* (0.60, 0.90)	<0.001
Overall image quality	3.19 ± 0.70	0.83* (0.65, 0.91)	3.26 ± 0.53	0.69 (0.39, 0.85)	2.53 ± 0.66	0.82* (0.65, 0.91)	4.73 ± 0.48	0.86* (0.72, 0.93)	<0.001

*Note*: Ratings are the average scores from 2 raters ± standard deviation. ICC data in parenthesis are 95% CIs.

* ICC values greater than 0.75 were considered as good agreement.

† P values were obtained by using Friedman tests to compare differences between four techniques.

Abbreviation: BH DW‐ss‐EPI, breath‐holding diffusion‐weighted single‐shot echo‐planar imaging, RT DW‐ss‐EPI, respiratory‐triggering diffusion‐weighted single‐shot echo‐planar imaging, FB DW‐ss‐EPI, free‐breathing diffusion‐weighted single‐shot echo‐planar imaging, FB DW‐Propeller‐EPI, free‐breathing diffusion‐weighted Propeller echo‐planar imaging, ICC, intraclass correlation coefficient.

**FIGURE 3:**
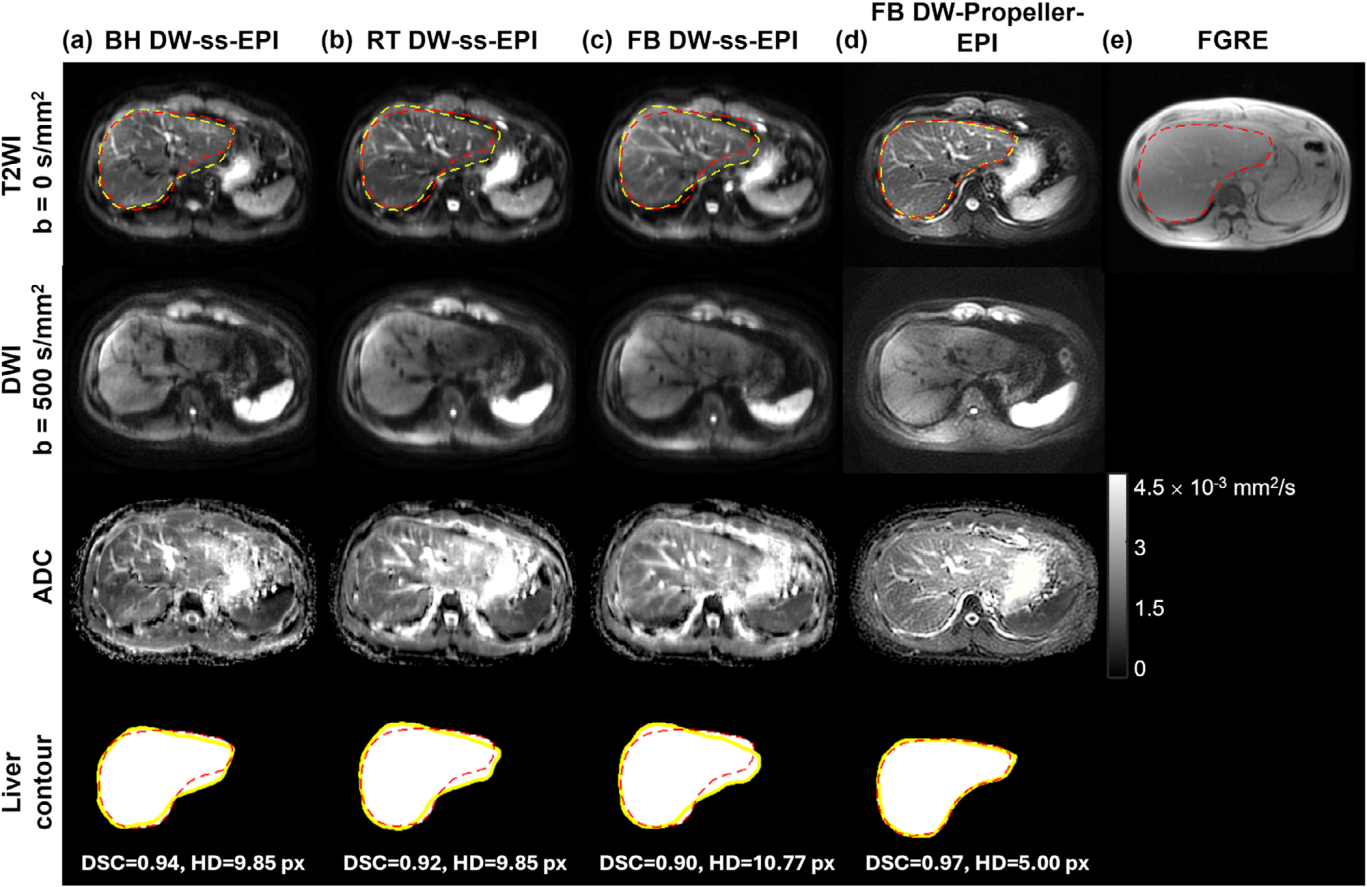
Representative liver diffusion images (at b‐values = 0 and 500 s/mm^2^) with corresponding ADC maps and obtained liver contours in a 30‐year‐old man, acquired using (a) breath‐holding diffusion‐weighted single‐shot echo‐planar imaging (BH DW‐ss‐EPI), (b) respiratory‐triggering diffusion‐weighted single‐shot echo‐planar imaging (RT DW‐ss‐EPI), (c) free‐breathing diffusion‐weighted single‐shot echo‐planar imaging (FB DW‐ss‐EPI), and (d) free‐breathing diffusion‐weighted Propeller echo‐planar imaging (FB DW‐Propeller‐EPI). The yellow dashed lines represent the liver contours from four techniques, and the red dashed lines indicate the gold‐standard liver contour obtained from (e) fast gradient echo (FGRE) image overlaid after centroid‐based registration. Dice Similarity Coefficient (DSC) and Hausdorff's Distance (HD) between the contours obtained from each technique and the FGRE image were calculated to quantitatively evaluate contour agreements. DWI images and ADC map obtained with FB DW‐Propeller‐EPI show superior image quality and geometric fidelity compared to the images obtained with the three DW‐ss‐EPI techniques. The liver contour from FB DW‐Propeller‐EPI had the largest DSC (0.97) and the smallest HD (5.00 px) values. The DWI image (on the second row) from FB DW‐Propeller‐EPI received higher ratings than the other 3 techniques across all five image quality aspects. Geometric fidelity scores (average of two raters) for BH DW‐ss‐EPI, RT DW‐ss‐EPI, FB DW‐ss‐EPI, and FB DW‐Propeller‐EPI techniques were: 3.5, 4, 3, and 5, respectively.

**FIGURE 4:**
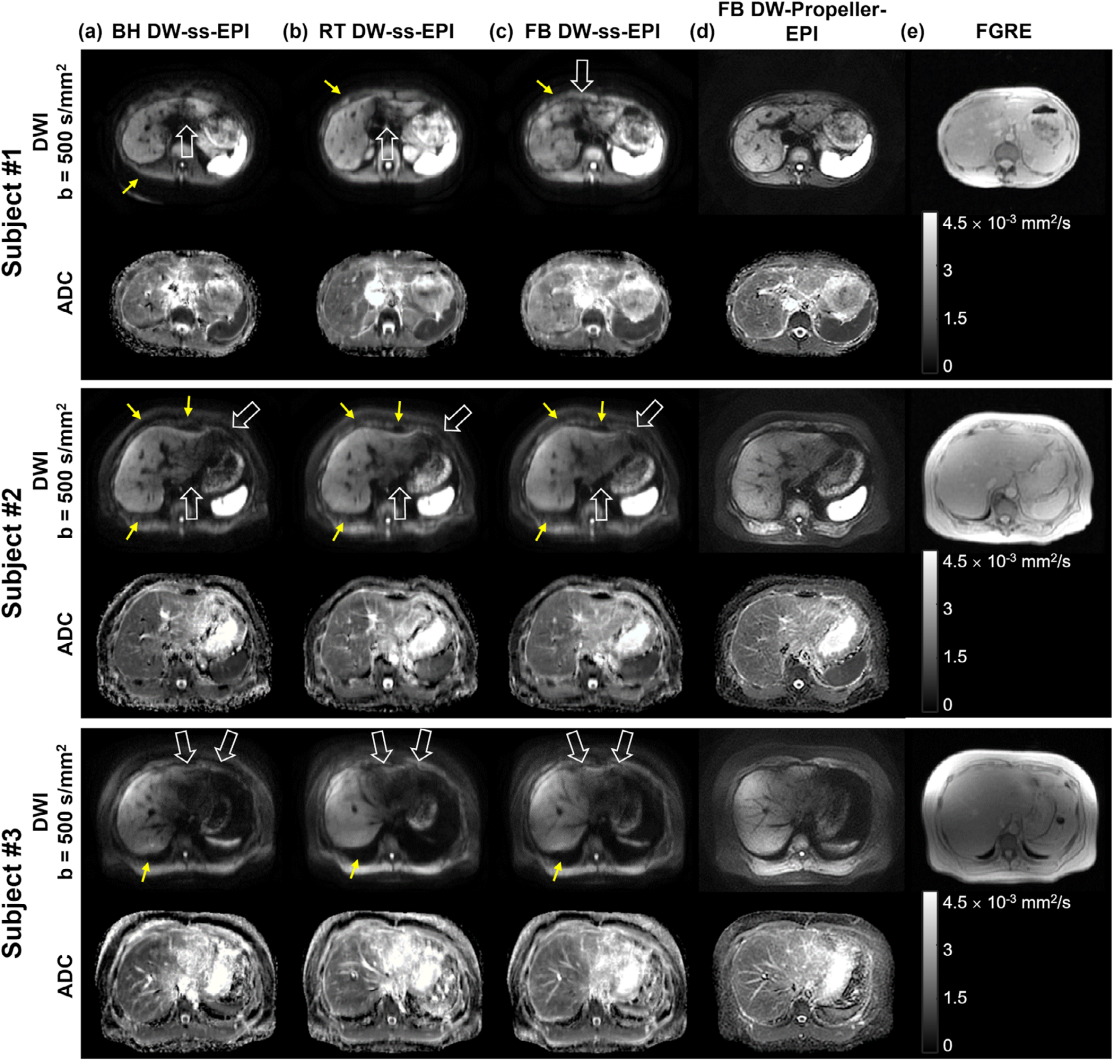
Liver diffusion images (at b‐value = 500 s/mm^2^) and corresponding ADC maps obtained from three subjects (Subject #1: a 51‐year‐old woman, Subject #2: a 63‐year‐old woman, and Subject #3: a 42‐year‐old man) are shown, acquired using four techniques: (a) breath‐holding diffusion‐weighted single‐shot echo‐planar imaging (BH DW‐ss‐EPI), (b) respiratory‐triggering diffusion‐weighted single‐shot echo‐planar imaging (RT DW‐ss‐EPI), (c) free‐breathing diffusion‐weighted single‐shot echo‐planar imaging (FB DW‐ss‐EPI), and (d) free‐breathing diffusion‐weighted Propeller echo‐planar imaging (FB DW‐Propeller‐EPI). (e) Fast gradient‐echo (FGRE) images, serving as the gold standard for geometric fidelity, are also displayed for each case. Yellow arrows highlight severer geometric distortion in DWI images obtained with three routine DW‐ss‐EPI techniques. In contrast, DWI images obtained with FB DW‐Propeller‐EPI technique exhibit geometric fidelity visually comparable to FGRE images. In addition, as indicated by the white arrows, DWI images obtained with three routine DW‐ss‐EPI techniques show more signal loss in the left liver lobe compared with FB DW‐Propeller‐EPI technique. For BH DW‐ss‐EPI, RT DW‐ss‐EPI, FB DW‐ss‐EPI and FB DW‐Propeller‐EPI techniques, average geometric fidelity scores from two raters were: 2, 3.5, 4, and 5 for subject #1; 3, 4, 2.5, and 5 for subject #2; 4, 3, 2, and 5 for subject #3. Average scores regarding signal homogeneity in the left lobe for the four techniques were: 3, 2, 4, and 5 for subject #1; 2, 3, 4, and 5 for subject #2; 3, 2, 3, and 4 for subject #3, respectively.

**FIGURE 5:**
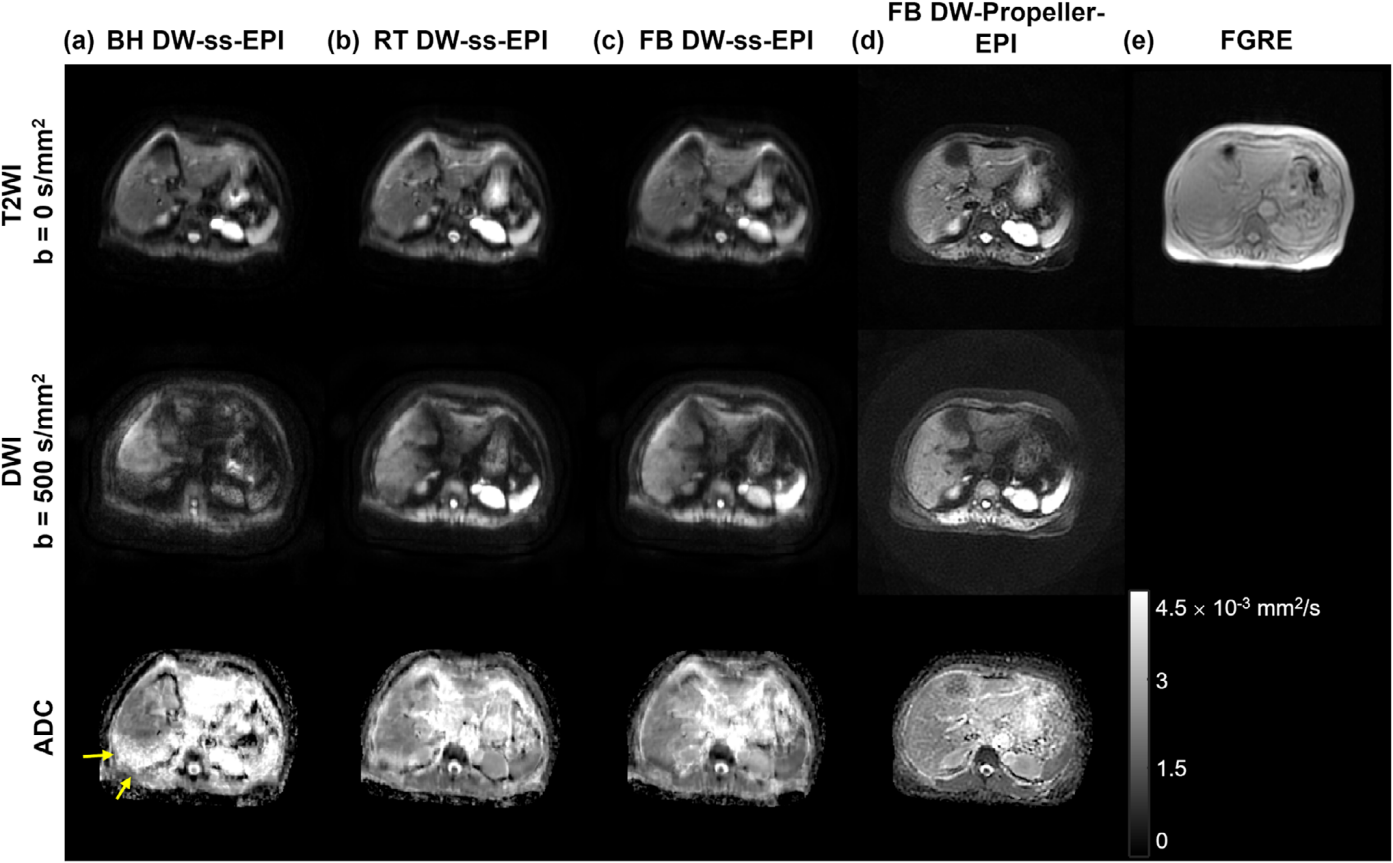
Representative liver diffusion images (at b‐value = 0 and 500 s/mm^2^) and corresponding ADC maps obtained from a 67‐year‐old woman with suboptimal breath holding, acquired using four techniques: (a) breath‐holding diffusion‐weighted single‐shot echo‐planar imaging (BH DW‐ss‐EPI), (b) respiratory‐triggering diffusion‐weighted single‐shot echo‐planar imaging (RT DW‐ss‐EPI), (c) free‐breathing diffusion‐weighted single‐shot echo‐planar imaging (FB DW‐ss‐EPI), and (d) free‐breathing diffusion‐weighted Propeller echo‐planar imaging (FB DW‐Propeller‐EPI). (e) Fast gradient‐echo (FGRE) images, serving as the gold standard for geometric fidelity. Yellow arrows indicate the prominent ADC calculation bias due to motion artifacts in the DWI image from the BH DW‐ss‐EPI technique. Based on evaluation by two radiologists, FB DW‐Propeller‐EPI yielded higher image quality scores than all three routine DW‐ss‐EPI techniques.

Among all rating aspects, the mean geometric fidelity score for FB DW‐Propeller‐EPI was the highest (4.86 ± 0.33), with 29 of 35 cases (83%) rated as excellent (i.e., no distortion) by both raters. In contrast, the mean geometric fidelity scores for the three routine DW‐ss‐EPI techniques ranged from 2.80 ± 0.70–3.40 ± 0.67, corresponding to ratings between severe and moderate distortion, and these ratings showed good interobserver agreement (ICC ranging from 0.78–0.81) (Table 3). Figures 3a–d demonstrate representative liver diffusion images (b‐values = 0 and 500 s/mm^2^) and corresponding ADC maps obtained with the four different techniques. The yellow dashed lines represent the liver contours from four techniques, and the red dashed lines show the gold‐standard liver contour obtained from fast gradient echo (FGRE) image (see example in Figure 3e) overlaid after centroid‐based registrations. FB DW‐Propeller‐EPI obtained the largest Dice's similarity coefficient (DSC) and the smallest Hausdorff's Distance (HD) values, which indicated its liver contour had the best overall spatial overlap and the minimum boundary deviation compared to the gold‐standard contour. Therefore, DWI images obtained using FB DW‐Propeller‐EPI showed superior image quality compared to the other routine DW‐ss‐EPI techniques, particularly regarding geometric fidelity. Three case examples presented in Figure 4 illustrate less geometric distortion (indicated by yellow arrows) and less signal loss in the left liver lobe (indicated by white arrows) in DWI images obtained with FB DW‐Propeller‐EPI. In addition, Figure 5 demonstrates that FB DW‐Propeller‐EPI can produce liver DWI images with acceptable image quality in a representative subject with poor breath‐holding ability, while images from DW‐ss‐EPI techniques show severe artifacts. Additional examples from two other such subjects are shown in Supplementary Figure S2. Tables S4– S6 in Supplementary Material summarize the average rating results for these three cases.

### 4.3 Repeatability of ADC measurements

**FIGURE 6:**
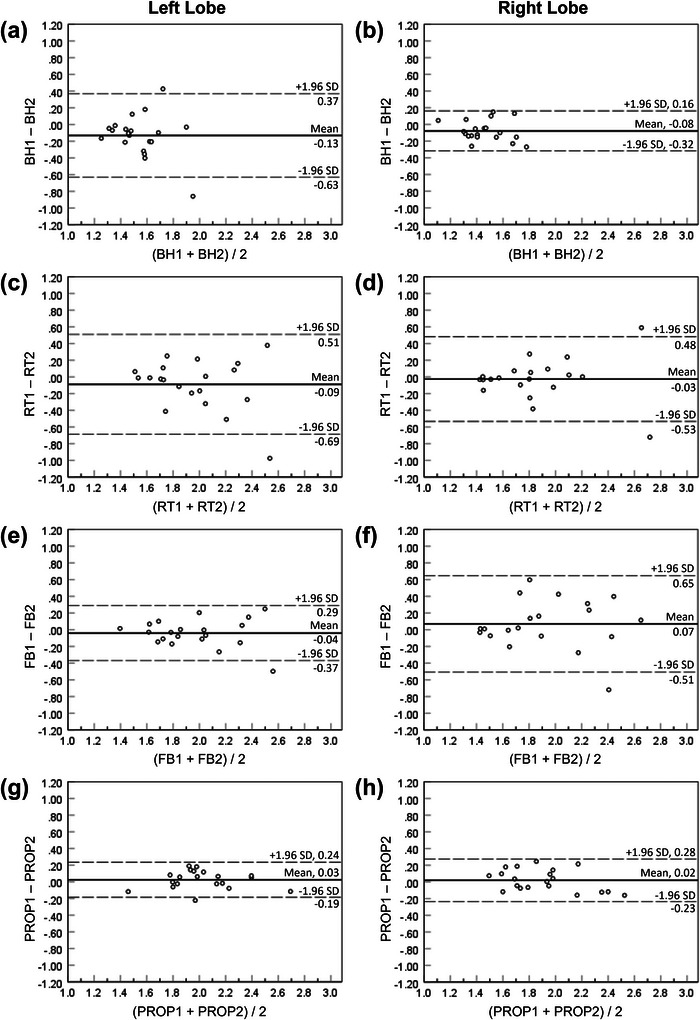
Bland‐Altman plots compare apparent diffusion coefficient (ADC) measurements between two repeated examinations in the left (a, c, e, g) and right (b, d, f, h) liver lobes with four techniques: (a, b) breath‐holding diffusion‐weighted single‐shot echo‐planar imaging (BH), (c, d) respiratory‐triggering diffusion‐weighted single‐shot echo‐planar imaging (RT), (e, f) free‐breathing diffusion‐weighted single‐shot echo‐planar imaging (FB), and (g, h) free‐breathing diffusion‐weighted Propeller echo‐planar imaging (PROP).

Table 4 and Figure 6 show the repeatability results of ADC measurements in the left and right liver lobes for the four techniques. Overall, FB DW‐Propeller‐EPI demonstrated the best repeatability for liver ADC measurements. In the left lobe, FB DW‐Propeller‐EPI showed notably better ADC repeatability (LOA = 0.212 × 10^−3^ mm^2^/s) compared to the other three routine DW‐ss‐EPI techniques (Table 4). In the right lobe, FB DW‐Propeller‐EPI and BH DW‐ss‐EPI exhibited comparably small LOAs (0.255 × 10^−3^ versus 0.241 × 10^−3^ mm^2^/s) and superior repeatability compared to the other two techniques (Table 4). Furthermore, the ADC repeatability of FB DW‐Propeller‐EPI was comparable between the left and right liver lobes (Table 4, Figure 6). In contrast, both BH DW‐ss‐EPI and RT DW‐ss‐EPI demonstrated worse ADC repeatability in the left liver lobe than that in the right lobe. Conversely, for FB DW‐ss‐EPI, the LOA in the left liver lobe was smaller than that in the right liver lobe, indicating better ADC repeatability in the left liver lobe for this technique.

**TABLE 4:** Repeatability of ADC measurements in the left and right liver lobes with four techniques.

Liver lobe	BH DW‐ss‐EPI (×10^−3^ mm^2^/s)	RT DW‐ss‐EPI (×10^−3^ mm^2^/s)	FB DW‐ss‐EPI (×10^−3^ mm^2^/s)	FB DW‐Propeller‐EPI (×10^−3^ mm^2^/s)
Left liver lobe	−0.130 ± 0.498	−0.088 ± 0.600	−0.040 ± 0.330	0.025 ± 0.212
Right liver lobe	−0.078 ± 0.241	−0.025 ± 0.507	0.071 ± 0.579	0.021 ± 0.255

*Note*: Data are mean difference ± 95% LOA.

Abbreviations: BH DW‐ss‐EPI, breath‐holding diffusion‐weighted single‐shot echo‐planar imaging; FB DW‐Propeller‐EPI, free‐breathing diffusion‐weighted Propeller echo‐planar imaging; FB DW‐ss‐EPI, free‐breathing diffusion‐weighted single‐shot echo‐planar imaging; RT DW‐ss‐EPI, respiratory‐triggering diffusion‐weighted single‐shot echo‐planar imaging.

### 4.4 Comparison of measured ADC values

**FIGURE 7:**
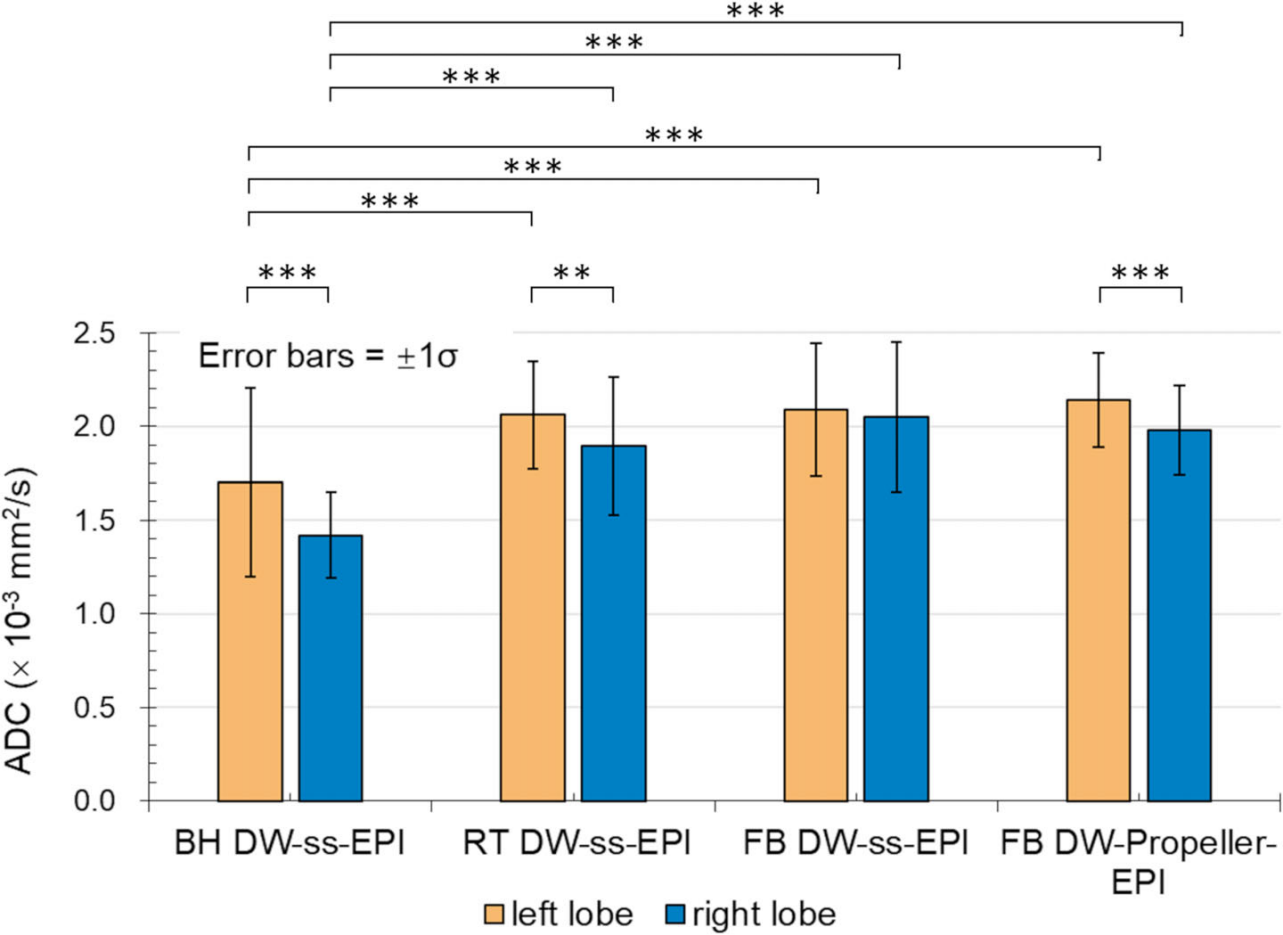
Bar plot shows mean apparent diffusion coefficients (ADCs) (± standard deviations) measured in the left and right liver lobes using four techniques. Error bars indicate ± 1 standard deviation (± 1σ). Statistical significance for comparisons between the left and right liver lobes for each technique and the pairwise comparisons among four techniques in either the left lobe or the right lobe are indicated with ** (P < 0.01) or *** (P < 0.001). BH DW‐ss‐EPI = breath‐holding diffusion‐weighted single‐shot echo‐planar imaging, RT DW‐ss‐EPI = respiratory‐triggering diffusion‐weighted single‐shot echo‐planar imaging, FB DW‐ss‐EPI = free‐breathing diffusion‐weighted single‐shot echo‐planar imaging, FB DW‐Propeller‐EPI = free‐breathing diffusion‐weighted Propeller echo‐planar imaging.

Figure 7 presents a bar plot of ADC values alongside the corresponding post‐hoc pairwise comparison results. ADC values measured in the left and right lobes using the four different techniques are summarized in Supplementary Table S7, while *P*‐values from the two‐way repeated measures ANOVA are presented in Supplementary Table S8. Significant differences in ADC measurements were found among the four techniques for both the left and right liver lobes (Supplementary Table S7). More specifically, BH DW‐ss‐EPI exhibited significantly lower ADC values (left lobe: 1.704 ± 0.503 × 10^−3^ mm^2^/s; right lobe: 1.420 ± 0.229 × 10^−3^ mm^2^/s) compared to the other three techniques in both the left and right liver lobes (Figure 7, Supplementary Table S8). Notably, no evidence suggested differences in ADC measurements among the RT DW‐ss‐EPI, FB DW‐ss‐EPI, and FB DW‐Propeller‐EPI techniques in either liver lobe (*P*‐value ranging from 0.11 to > 0.99) (Supplementary Table S8). When comparing ADC measurements between the left and right lobes, no significant difference was found for FB DW‐ss‐EPI (*P* = 0.53) (Supplementary Table S7), whereas significantly higher mean ADC values were observed in the left lobe compared to the right lobe for the other three techniques.

### 4.5 Effects of the number of blades on FB DW‐propeller‐EPI performance

**FIGURE 8:**
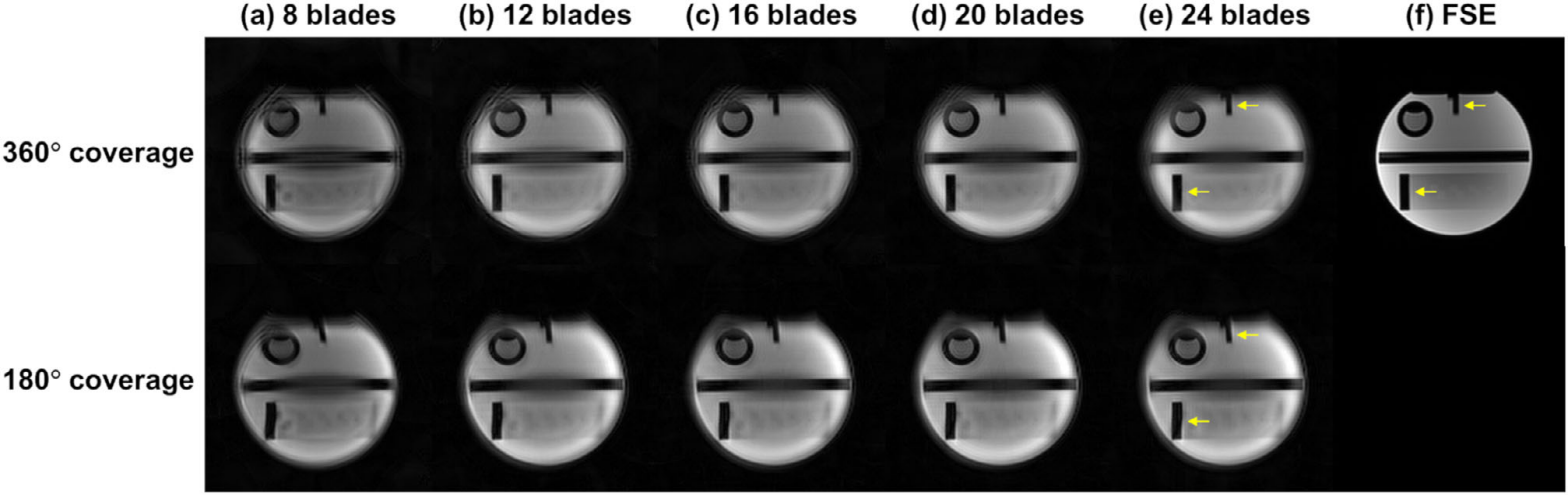
Phantom T2WI images reconstructed from data acquired with two different k‐space coverages (360° and 180°) and five different numbers of blades: (a) 8 blades, (b) 12 blades, (c) 16 blades, (d) 20 blades, and (e) 24 blades. (f) FSE image was acquired as reference. When the number of blades is held constant, images acquired with a 360° k‐space coverage show better suppression of image blurring and distortion associated with the off‐resonance effect in each blade. However, 360° k‐space coverage requires more blades to retain sufficient angular sampling density and minimize streaking artifacts. Fewer blades lead to more obvious streaking artifacts.

Figure 8 shows the comparison between phantom images reconstructed from 10 datasets and the reference fast spin echo (FSE) image. When the number of blades was held constant, 360° coverage consistently outperformed 180° coverage in preserving structural fidelity. As the yellow arrows indicate, the image acquired using 180° coverage exhibited noticeable residual distortion in fine anatomical features. In contrast, 360° coverage with an adequate number of blades demonstrated significantly reduced blurring and distortion. In addition, fewer blades led to more obvious streaking artifacts. While 360° coverage offered clear advantages, when the blade count was reduced to 12 blades, pronounced streaking artifacts existed in the reconstructed images.

**FIGURE 9:**
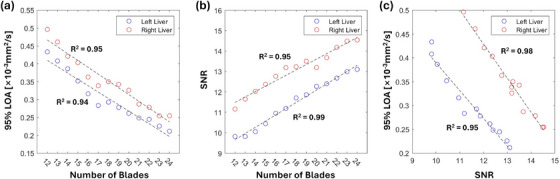
Effects of the number of blades on ADC repeatability and SNR in Free‐breathing (FB) Liver DW‐Propeller‐EPI. (a) Highly correlated line‐fitting graphs of number of blades and ADC repeatability. (b) Highly correlated line‐fitting graphs of number of blades and SNR. (c) The strong correlation between SNR and improve ADC repeatability, with comparable performance between the left and right liver lobes.

Figure 9 illustrates the relationship of the number of blades with ADC repeatability and SNR. The calculated ADC repeatability and SNR values from images reconstructed with various numbers of blades are shown in Supplementary Table 9. For both liver lobes, ADC repeatability and SNR exhibited linear increments with the number of blades. It is noticeable that when using only 12 blades for data reconstruction, the effective acquisition time of FB DW‐Propeller‐EPI (3.2 min) can be comparable to RT DW‐ss‐EPI, while with slightly better ADC repeatability (supplementary Table 9). In addition, with the same number of blades, the calculated LOAs were comparable for the left and right liver lobes.

## 5 DISCUSSION

This study aimed to develop and evaluate an advanced FB liver DWI technique based on a multi‐shot DWI technique, namely DW‐Propeller‐EPI acquisition. An integrated reconstruction pipeline was developed to achieve robust Nyquist ghost correction and reliable data reconstruction for the liver DWI data acquired during FB. In all qualitative evaluation aspects, the proposed technique enabled FB liver DWI with significantly superior image quality to three routine liver DWI techniques. Furthermore, in quantitative evaluation, FB DW‐Propeller‐EPI demonstrated high repeatability of ADC measurements, with comparable performance between the left and right liver lobes.

In routine liver DWI, the use of BH leads to a short scan time but limits the achievable image resolution and SNR. Furthermore, BH may not be feasible for patients with limited BH capability or those unable to follow the instructions to complete the BH (e.g., sick, elderly, or pediatric patients). Incomplete BH, in turn, may lead to pronounced motion artifacts, which can affect the accuracy of liver ADC measurements. In addition, when applying respiratory triggering to abdominal MRI examinations, sick or stressed patients may exhibit irregular or heavy breathing. This can prolong the total scan time^31^ and potentially introduce errors in liver ADC measurements due to pseudo‐anisotropy artifacts.^6^, ^32^ Accordingly, there has been increasing interest in developing FB abdominal MRI techniques for patients unable to cooperate. While multi‐averaged FB DWI is more SNR‐efficient and better suited for challenging populations than RT methods, the resulting DW images can exhibit blurring due to data misregistration during volume averaging.^10^, ^33^ Nevertheless, a considerable number of FB techniques based on radial sampling have been developed for liver fat measurement^34^, ^35^, ^36^, ^37^ or dynamic liver imaging.^38^, ^39^ For FB liver DWI, the DW‐Propeller‐EPI technique proposed in this study demonstrated satisfactory performance in terms of image quality and ADC repeatability. This suggests that it may serve as a better alternative to routine FB liver DWI technique. The reduced susceptibility to respiratory motion observed in FB DW‐Propeller‐EPI is likely attributable to its rotating multi‐blade acquisition, which leverages the advantages of a radial sampling scheme.^31^, ^39^ Notably, similar motion reduction has also been observed in brain studies,^24^, ^26^, ^29^ further supporting the motion compensation capability of DW‐Propeller‐EPI.

In the qualitative evaluation, DW‐Propeller‐EPI demonstrated significantly lower image distortion, even without the use of parallel imaging for each blade. To ensure a fair comparison, the three routine liver DWI techniques were also performed without parallel imaging. As a result, all four techniques experienced a similar extent of off‐resonance effects due to their comparable EPI echo spacings. The capability of DW‐Propeller‐EPI to reduce geometric distortion in liver DWI aligns with findings from previous brain DWI studies.^24^, ^26^, ^29^ These studies suggest that the rotating blades in Propeller‐EPI can distribute off‐resonance effect across different phase‐encoding directions, thereby minimizing geometric distortion. The improved geometric fidelity of liver DWI using DW‐Propeller‐EPI may provide particular advantages for MRI‐guided abdominal interventions^40^ and radiation therapy.^41^ This is especially relevant because DWI has been shown to accurately delineate the tumor without contrast injection for treatment planning and monitoring,^42^ and DW‐Propeller‐EPI may substantially reduce localization errors caused by image distortion during procedural guidance.

Compared to conventional DW‐ss‐EPI techniques, the FB liver DW‐Propeller‐EPI also yielded improvements in signal retention in the left liver lobe, liver edge sharpness, and vessel clarity. The performance of FB DW‐Propeller‐EPI on subjects who had suboptimal breath holding further supports its advantage in dealing with motion issues. These improvements were likely attributable to the reconstruction framework, the reduced echo train length (ETL) in each blade in DW‐Propeller‐EPI, and the motion compensation capability of DW‐Propeller‐EPI. The reconstruction framework can effectively minimize ghost artifacts and stabilize signal variations across multiple blades via correlation weighting,^14^ thus reducing the influence of signal variations and dropouts present in some blades. The reference‐free ghost correction method is essential for liver DWI under FB conditions, not only to eliminate the extra calibration time required for each blade, but also to enhance image quality, as correction parameters derived from another reference image may introduce residual artifacts (as shown in Supplementary Figure S3). In addition, compared to DW‐ss‐EPI, the reduced ETL in each blade of FB DW‐Propeller‐EPI can mitigate image blurring from the T2* windowing effect, and likely contributed to the improved liver edge sharpness and the vessel clarity. Furthermore, the multi‐blade data combination in DW‐Propeller‐EPI could also leverage signal averaging to enhance motion robustness for FB liver DWI. Previous liver DWI studies indicated that increasing the number of signal averages can alleviate signal loss in the left liver lobe and improve the repeatability of ADC measurements.^11^, ^43^ Results from our phantom study and retrospective reconstructions indicate that incorporating more blades and employing 360° k‐space coverage during reconstruction can improve geometric fidelity, image SNR, and ADC repeatability. This suggests that with a sufficient number of blades, DW‐Propeller‐EPI can achieve satisfactory image quality and ADC repeatability under FB condition. However, caution must be exercised, as shortening total scan time by reducing the number of blades comes at the cost of degraded image quality and reduced ADC repeatability.

While liver DWI shows notable promise for diagnosis using ADC measurement, the presence of image artifacts in DW‐ss‐EPI can reduce the reliability and repeatability of measured ADC values,^6^ thereby limiting its clinical use. Particularly, the ADC repeatability in the left lobe is often worse than that in the right lobe because of the cardiac motions.^11^ In our study, discrepancy in ADC repeatability between two liver lobes was similarly observed across three routine liver DW‐ss‐EPI techniques. In contrast, FB DW‐Propeller‐EPI not only demonstrated better ADC repeatability compared to DW‐ss‐EPI techniques, but also achieved comparable ADC repeatability between left and right liver lobes. The improvement in liver ADC repeatability could be attributed to a combination of the improved motion robustness, increased image quality (especially the better signal retention in left liver lobe), and signal averaging of multi‐blades data. In the case of FB DW‐Propeller‐EPI, the improved ADC repeatability observed in both left and right liver lobes suggests a potential advancement in the clinical feasibility of liver ADC measurement, such as for lesion characterization and longitudinal monitoring of treatment response. Importantly, the comparable ADC repeatability in the left liver lobe indicates that FB DW‐Propeller‐EPI may enable more reliable investigations of liver diseases in this region. Furthermore, given that no significant differences were found in measured ADC values between FB DW‐Propeller‐EPI and routine RT or FB DW‐ss‐EPI techniques, the ADC thresholds established for RT or FB DW‐ss‐EPI can be directly adopted for currently proposed clinical applications.

This study has several limitations. First, DW‐Propeller‐EPI is a multi‐shot technique that requires multiple acquisitions to fill k‐space, resulting in inherently longer scan time compared to DW‐ss‐EPI techniques. As the scan time of FB liver DW‐Propeller‐EPI (6.4 min) demonstrated in this work has nearly reached the clinically acceptable limit, future studies should explore ways to reduce acquisition time. For example, imaging acceleration techniques, such as simultaneous multi‐slice acceleration,^9^, ^44^ can be incorporated to improve scan efficiency. In addition, further optimization of the number of blades may help reduce scan time. In this study, FB DW‐Propeller‐EPI with 12 blades demonstrated the potential to outperform RT DW‐ss‐EPI in ADC repeatability within a comparable scan time (3.2 min), although the k‐space distributions of the blades were limited by the retrospective undersampling approach. Additionally, the phantom study results for quality assessment further highlighted the key trade‐off between reduced acquisition time (fewer blades) and overall image quality in Propeller‐EPI reconstructions (Figure 8). Therefore, further optimization of the number and the k‐space distributions of blades in FB DW‐Propeller‐EPI is needed and may greatly enhance the clinical feasibility of this technique. Future work may also involve the use of motion‐compensated diffusion‐encoding gradients, which can further enhance the motion robustness of FB liver DW‐Propeller‐EPI,^45^ potentially reducing the required number of blades while maintaining high ADC repeatability. Second, to facilitate comparison of ADC repeatability during clinical examinations, the scan parameters for the three routine DW‐ss‐EPI techniques were selected from common clinical protocols. As a result, differences in scan time and signal averaging existed among the four techniques. Third, future studies are needed to assess the performance of FB liver DW‐Propeller‐EPI with parallel imaging, particularly in improving image quality. Fourth, future study could also compare FB DW‐Propeller‐EPI with other multi‐shot techniques, such as MUSE, to evaluate relative performance in reducing motion artifacts and optimizing acquisition efficiency. Fifth, FB liver DW‐Propeller‐EPI was tested only with a b‐value of 500 s/mm^2^ on a 1.5T MRI scanner. Future investigations are needed to evaluate its performance across a broader range of b‐values and at different field strengths, such as 3.0 T. In addition, due to the limited number of recruited subjects with focal hepatic lesions in our cohort, no analysis of liver lesions was performed in this study. Future work is warranted to further evaluate the performance of FB DW‐Propeller‐EPI in lesion detection. Finally, future research should evaluate the clinical applicability of FB DW‐Propeller‐EPI in elderly and pediatric populations, particularly under challenging clinical scenarios.

## 6 CONCLUSIONS

The FB DW‐Propeller‐EPI technique effectively enabled FB liver DWI, demonstrating superior image quality and improved ADC repeatability compared to routine liver DW‐ss‐EPI techniques. This finding suggests that with a sufficient number of blades, FB DW‐Propeller‐EPI may serve as a valuable alternative for routine FB liver DWI protocols, particularly benefiting challenging patients requiring such protocols for clinical diagnosis based on ADC measurement.

## CONFLICT OF INTEREST STATEMENT

The authors have no conflicts to disclose.

## Supporting information

Supporting Information

## Data Availability

The data that support the findings of this study are available from the corresponding author upon reasonable request.
