# Supplementary material for: Advancing free‐breathing liver diffusion‐weighted imaging with Propeller‐EPI: Improved image quality and ADC repeatability
Source: Med Phys. 2025 Dec 4;52(12):e70180. doi: 10.1002/mp.70180 (PMC12678224; doi:10.1002/mp.70180)
Supplement: Supplementary file 1 — Supporting Information [file MP-52-0-s001.docx]

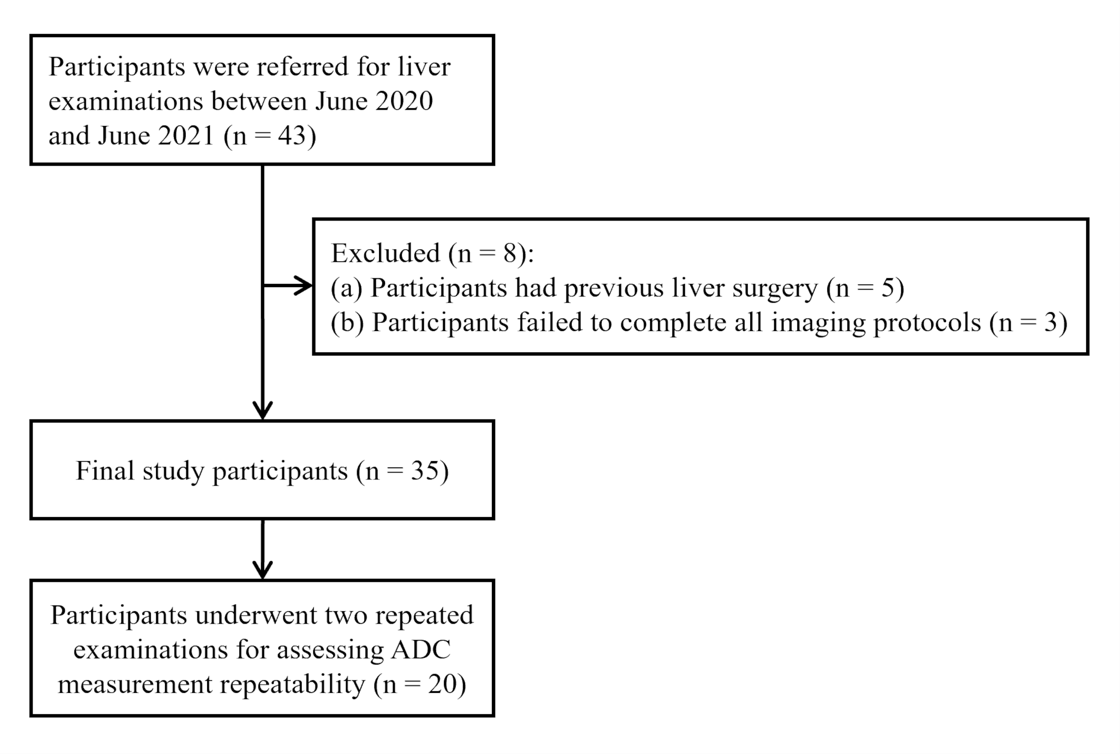


**Supplementary Figure S1.** Study inclusion and exclusion flowchart. ADC = apparent diffusion coefficient.


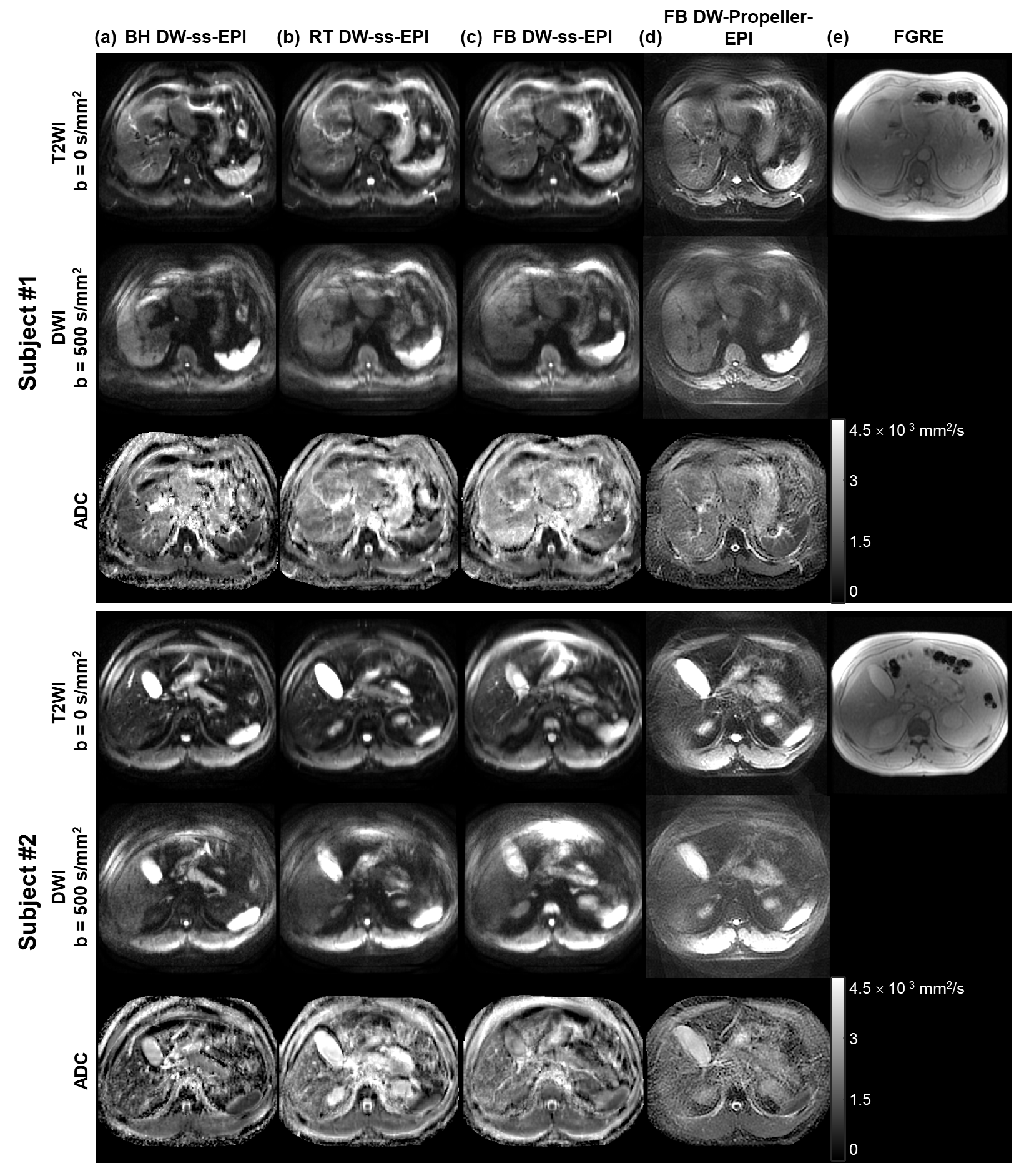


**Supplementary Figure S2.** Representative liver diffusion images (at b-value = 0 and 500 s/mm^2^) and corresponding ADC maps obtained from two subjects (Subject #1: a 57-year-old man and Subject #2: a 40-year-old man) with suboptimal breath holding are shown, acquired using four techniques: (**a**) breath-holding diffusion-weighted single-shot echo-planar imaging (BH DW-ss-EPI), (**b**) respiratory-triggering diffusion-weighted single-shot echo-planar imaging (RT DW-ss-EPI), (**c**) free-breathing diffusion-weighted single-shot echo-planar imaging (FB DW-ss-EPI), and (**d**) free-breathing diffusion-weighted Propeller echo-planar imaging (FB DW-Propeller-EPI). **(e)** Fast gradient-echo (FGRE) images, serving as the gold standard for geometric fidelity. Subject #1 failed to hold his breath and was excluded in this study while subjects #2 had suboptimal breath holding.

**
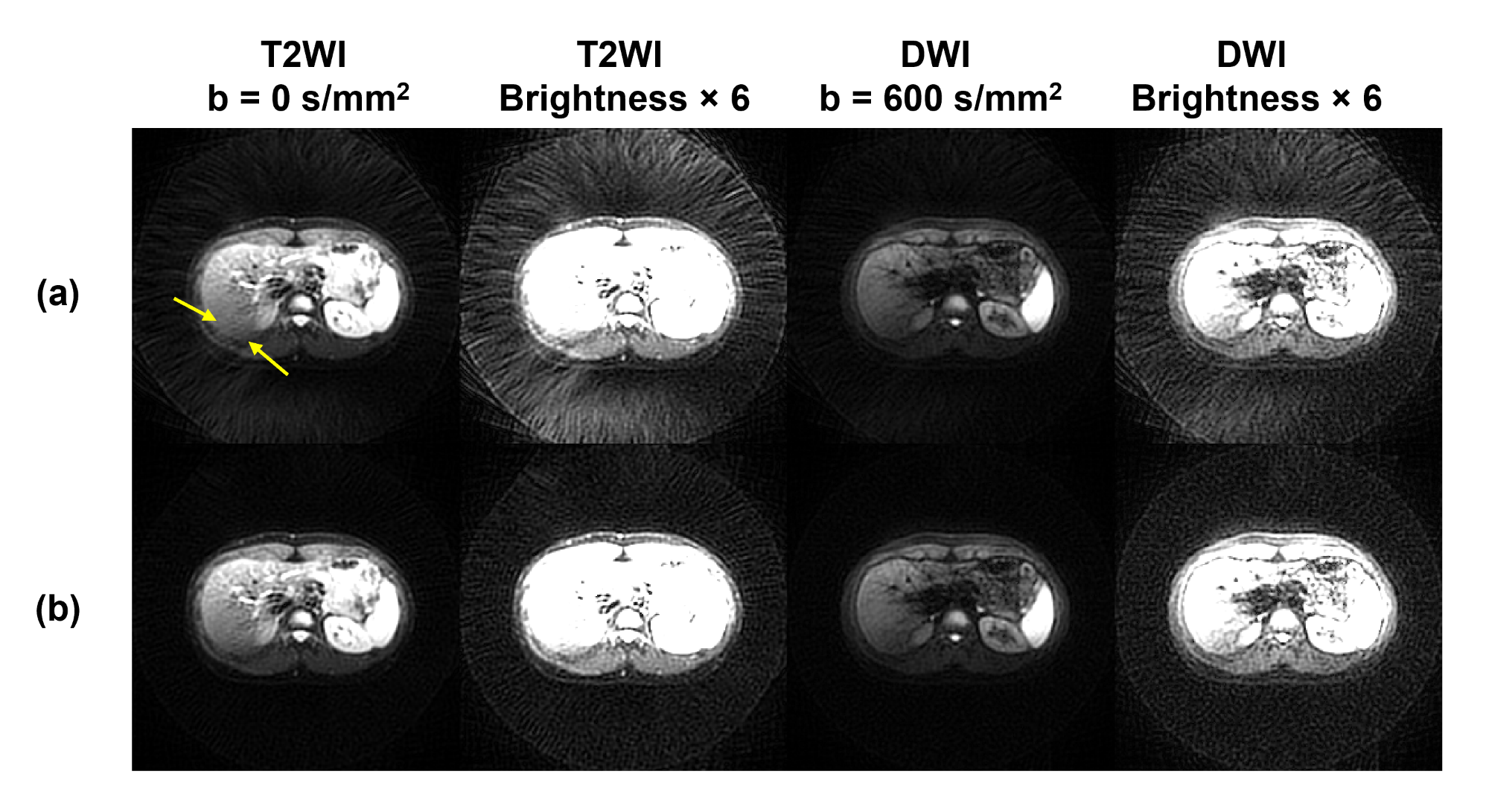
**

**Supplementary Figure S3.** Representative liver diffusion images (at b-values of 0 and 600 s/mm²) in a 28-year-old male volunteer, reconstructed using the DW-Propeller-EPI reconstruction pipeline with ghost correction parameters derived from **(a)** the T2WI image of a selected reference slice with the largest anatomical region and **(b)** the T2WI image of the same slice. The acquisition parameters included: FOV = 400 mm, reconstruction matrix = 128 × 128, blade size = 128 × 32, slice thickness = 8 mm, number of blades = 18, k-space coverage = 360°. Images corrected using parameters from another slice exhibited obvious residual artifacts in both the liver parenchyma (indicated by yellow arrows) and the background, while ghost correction using parameters derived from the same slice effectively reduced the artifacts. This suggests that effective 2D ghost correction for all blades at each slice location is crucial for achieving optimal image quality in DW-Propeller-EPI under free-breathing conditions. Moreover, although several reference-based correction methods can also effectively eliminate ghost artifacts associated with oblique or double-oblique scans, these methods require longer scan time to collect reference data for each blade at each slice location.

**Supplementary Table S1. Scan parameters for phantom experiments with five different numbers of blades for sampling k-space with two different coverages.**

| The number of blades | 8 | 12 | 16 | 20 | 24 |
| --- | --- | --- | --- | --- | --- |
| Blade size | 128 × 50 | 128 × 32 | 128 × 32 | 128 × 32 | 128 × 32 |
| TE (ms) | 76.4 | 66 | 66 | 66 | 66 |
| Scan time (s)* | 64 | 96 | 128 | 160 | 192 |
| Rotating angle for 360° coverage (degrees) | 45 | 30 | 22.5 | 18 | 15 |
| Rotating angle for 180° coverage (degrees) | 22.5 | 15 | 11.25 | 9 | 7.5 |

* The scan time is for acquiring DWI images with one diffusion direction and two b-values (0 and 500 s/mm^2^).

**Supplementary Table S2**

Demographic Characteristics.

| Group and Variable | Value |
| --- | --- |
| All participants (*n* = 35) | |
| Age (y)* | 46 ± 15 |
| Age range (y) | 22–72 |
| Sex |  |
| Male | 24/35 (69) |
| Female | 11/35 (31) |
| Participants underwent repeated acquisitions for assessing ADC measurement repeatability (*n* = 20) | |
| Age (y)* | 41 ± 15 |
| Age range (y) | 22–66 |
| Sex |  |
| Male | 12/20 (60) |
| Female | 8/20 (40) |

Note. Unless otherwise specified, data are numbers of participants, with percentages in parentheses.

* Data are mean ± SDs.

| **Supplementary Table S3. P values from post-hoc pairwise tests of Friedman test for qualitative image quality comparison** | | | | | | |
| --- | --- | --- | --- | --- | --- | --- |
| **Rating Aspects** | **BH DW-ss-EPI vs**  **RT DW-ss-EPI** | **BH DW-ss-EPI vs**  **FB DW-ss-EPI** | **BH DW-ss-EPI vs**  **FB DW-Propeller-EPI** | **RT DW-ss-EPI vs**  **FB DW-ss-EPI** | **RT DW-ss-EPI vs**  **FB DW-Propeller-EPI** | **FB DW-ss-EPI vs**  **FB DW-Propeller-EPI** |
| Signal homogeneity in the left liver lobe | >0.99 | 0.38 | <0.001* | >0.99 | <0.001* | <0.001* |
| Geometric fidelity | 0.20 | >0.99 | <0.001* | >0.99 | <0.001* | <0.001* |
| Liver edge sharpness | >0.99 | 0.22 | <0.001* | >0.99 | <0.001* | <0.001* |
| Vessel clarity | >0.99 | 0.07 | <0.001* | 0.01* | <0.001* | <0.001* |
| Overall image quality | >0.99 | 0.14 | <0.001* | 0.03* | <0.001* | <0.001* |
| P values were obtained from post-hoc pairwise comparisons with Bonferroni correction. BH DW-ss-EPI, breath-holding diffusion-weighted single-shot echo-planar imaging; RT DW-ss-EPI, respiratory-triggering diffusion-weighted single-shot echo-planar imaging; FB DW-ss-EPI, free-breathing diffusion-weighted single-shot echo-planar imaging; FB DW-Propeller-EPI, free-breathing diffusion-weighted Propeller echo-planar imaging. | | | | | | |

**Supplementary Table S4.** Qualitative image quality ratings for subject #1 who failed to hold his breath

| **Subject #1** | **BH DW-ss-EPI** | **RT DW-ss-EPI** | **FB DW-ss-EPI** | **FB DW-Propeller-EPI** |
| --- | --- | --- | --- | --- |
| Signal homogeneity in the left liver lobe | 2 | 2 | 3 | 5 |
| Geometric fidelity | 2 | 2 | 2 | 5 |
| Liver edge sharpness | 1.5 | 1.5 | 1.5 | 5 |
| Vessel clarity | 2 | 2 | 2 | 4.5 |
| Overall image quality | 2 | 2 | 2 | 5 |

**Supplementary Table S5.** Qualitative image quality ratings for subject #2 who had bad breath holding

| **Subject #2** | **BH DW-ss-EPI** | **RT DW-ss-EPI** | **FB DW-ss-EPI** | **FB DW-Propeller-EPI** |
| --- | --- | --- | --- | --- |
| Signal homogeneity in the left liver lobe | 3 | 2 | 1.5 | 4 |
| Geometric fidelity | 2 | 2.5 | 2.5 | 4.5 |
| Liver edge sharpness | 2 | 1 | 1.5 | 4 |
| Vessel clarity | 2.5 | 1 | 1 | 3.5 |
| Overall image quality | 2.5 | 2 | 1.5 | 4 |

**Supplementary Table S6.** Qualitative image quality ratings for subject #3 who had bad breath holding.

| **Subject #3** | **BH DW-ss-EPI** | **RT DW-ss-EPI** | **FB DW-ss-EPI** | **FB DW-Propeller-EPI** |
| --- | --- | --- | --- | --- |
| Signal homogeneity in the left liver lobe | 1 | 1.5 | 2 | 5 |
| Geometric fidelity | 1.5 | 2 | 2 | 5 |
| Liver edge sharpness | 1.5 | 2.5 | 2.5 | 5 |
| Vessel clarity | 1 | 1.5 | 2 | 4 |
| Overall image quality | 1 | 2 | 2 | 5 |

| **Supplementary Table S7. ADC measurements in left and right liver lobes with four techniques and comparison results from two-way repeated measures ANOVA** | | | | | |
| --- | --- | --- | --- | --- | --- |
|  | **BH DW-ss-EPI (× 10^-3^ mm^2^/sec)** | **RT DW-ss-EPI (× 10^-3^ mm^2^/sec)** | **FB DW-ss-EPI (× 10^-3^ mm^2^/sec)** | **FB DW-Propeller-EPI (× 10^-3^ mm^2^/sec)** | **P value*** |
| Location |  |  |  |  |  |
| Left lobe | 1.704 ± 0.503 | 2.061 ± 0.289 | 2.090 ± 0.353 | 2.139 ± 0.253 | <0.001 |
| Right lobe | 1.420 ± 0.229 | 1.898 ± 0.369 | 2.051 ± 0.402 | 1.980 ± 0.240 | <0.001 |
| P value† | <0.001 | 0.004 | 0.53 | <0.001 | — |
| Data are mean ± standard deviation. BH DW-ss-EPI, breath-holding diffusion-weighted single-shot echo-planar imaging; RT DW-ss-EPI, respiratory-triggering diffusion-weighted single-shot echo-planar imaging; FB DW-ss-EPI, free-breathing diffusion-weighted single-shot echo-planar imaging; FB DW-Propeller-EPI, free-breathing diffusion-weighted Propeller echo-planar imaging.  * Comparison of ADC values between four techniques.  † Comparison of ADC values between left and right liver lobes for each technique | | | | | |

| **Supplementary Table S8. P values from post-hoc pairwise comparisons of two-way repeated measures ANOVA** | | | | | | | | | | |
| --- | --- | --- | --- | --- | --- | --- | --- | --- | --- | --- |
|  | **Between Techniques*** | | | | | | | | | **Between Lobes†** |
|  | **Left lobe** | | | | **Right Lobe** | | | | |  |
| Techniques | BH DW-ss-EPI | RT DW-ss-EPI | FB DW-ss-EPI | FB DW-Propeller-EPI | | BH DW-ss-EPI | RT DW-ss-EPI | FB DW-ss-EPI | FB DW-Propeller-EPI | Left lobe vs Right lobe |
| BH DW-ss-EPI | — | — | — | — | | — | — | — | — | <0.001 |
| RT DW-ss-EPI | <0.001 | — | — | — | | <0.001 | — | — | — | 0.004 |
| FB DW-ss-EPI | <0.001 | >0.99 | — | — | | <0.001 | 0.11 | — | — | 0.53 |
| FB DW-Propeller-EPI | <0.001 | 0.49 | >0.99 | — | | <0.001 | 0.48 | >0.99 | — | <0.001 |
| BH DW-ss-EPI, breath-holding diffusion-weighted single-shot echo-planar imaging; RT DW-ss-EPI, respiratory-triggering diffusion-weighted single-shot echo-planar imaging; FB DW-ss-EPI, free-breathing diffusion-weighted single-shot echo-planar imaging; FB DW-Propeller-EPI, free-breathing diffusion-weighted Propeller echo-planar imaging.  * Pairwise Comparison of ADC values between four techniques.  † Pairwise Comparison of ADC values between left and right liver lobes for each technique. | | | | | | | | | | |

|  | **Left Liver Lobe** | | **Right Liver Lobe** | |
| --- | --- | --- | --- | --- |
| **Number of blades** | **Mean Difference ± 95% LOA**  **(× 10^-3^ mm^2^/s)** | **SNR** | **Mean Difference ± 95% LOA**  **(× 10^-3^ mm^2^/s)** | **SNR** |
| 12 | -0.011 ± 0.433 | 9.818 | 0.052 ± 0.496 | 11.160 |
| 13 | -0.006 ± 0.408 | 9.814 | 0.055 ± 0.461 | 11.646 |
| 14 | -0.007 ± 0.386 | 10.053 | 0.064 ± 0.421 | 12.025 |
| 15 | -0.006 ± 0.352 | 10.445 | 0.061 ± 0.404 | 12.371 |
| 16 | 0.002 ± 0.316 | 10.954 | 0.052 ± 0.363 | 12.765 |
| 17 | 0.008 ± 0.283 | 11.192 | 0.042 ± 0.339 | 13.189 |
| 18 | 0.009 ± 0.293 | 11.705 | 0.031 ± 0.350 | 13.233 |
| 19 | 0.017 ± 0.278 | 11.860 | 0.032 ± 0.343 | 13.502 |
| 20 | 0.014 ± 0.261 | 12.271 | 0.036 ± 0.326 | 13.191 |
| 21 | 0.009 ± 0.248 | 12.398 | 0.017 ± 0.287 | 13.672 |
| 22 | 0.010 ± 0.245 | 12.675 | 0.015 ± 0.279 | 14.182 |
| 23 | 0.018 ± 0.226 | 12.991 | 0.021 ± 0.254 | 14.488 |
| 24 | 0.025 ± 0.212 | 13.103 | 0.021 ± 0.255 | 14.539 |

**Supplementary Table S9. Influence of the number of blades on ADC repeatability in Free-breathing (FB) Liver DW-Propeller-EPI.**
